# PANATIKI: A Network Access Control Implementation Based on PANA for IoT Devices

**DOI:** 10.3390/s131114888

**Published:** 2013-11-01

**Authors:** Pedro Moreno Sanchez, Rafa Marin Lopez, Antonio F. Gomez Skarmeta

**Keywords:** IoT, network access control, PANA, EAP, AAA, light-weight

## Abstract

Internet of Things (IoT) networks are the pillar of recent novel scenarios, such as smart cities or e-healthcare applications. Among other challenges, these networks cover the deployment and interaction of small devices with constrained capabilities and Internet protocol (IP)-based networking connectivity. These constrained devices usually require connection to the Internet to exchange information (e.g., management or sensing data) or access network services. However, only authenticated and authorized devices can, in general, establish this connection. The so-called authentication, authorization and accounting (AAA) services are in charge of performing these tasks on the Internet. Thus, it is necessary to deploy protocols that allow constrained devices to verify their credentials against AAA infrastructures. The *Protocol for Carrying Authentication for Network Access* (PANA) has been standardized by the Internet engineering task force (IETF) to carry the *Extensible Authentication Protocol* (EAP), which provides flexible authentication upon the presence of AAA. To the best of our knowledge, this paper is the first deep study of the feasibility of EAP/PANA for network access control in constrained devices. We provide light-weight versions and implementations of these protocols to fit them into constrained devices. These versions have been designed to reduce the impact in standard specifications. The goal of this work is two-fold: (1) to demonstrate the feasibility of EAP/PANA in IoT devices; (2) to provide the scientific community with the first light-weight interoperable implementation of EAP/PANA for constrained devices in the Contiki operating system (Contiki OS), called PANATIKI. The paper also shows a testbed, simulations and experimental results obtained from real and simulated constrained devices.

## 1. Introduction

The growth of small devices with constrained capabilities and *Internet Protocol* (IP)-based networking connectivity is today a reality. They typically form self-configurable wireless multi-hop networks of relay nodes, which are able to recover from communication failures. Due to these features, they have become an important part of the Smart Grid, as well as sensor networks, such as the Internet of Things. ZigBee [1] is a typical example of multi-hop networking technology based on the IEEE 802.15.4 [2] standard, which uses *IP version 6 over Low power Wireless Personal Area Networks* (6LoWPAN) [3] to integrate *IP version 6* (IPv6)-based connectivity in constrained devices.

**Figure 1.**
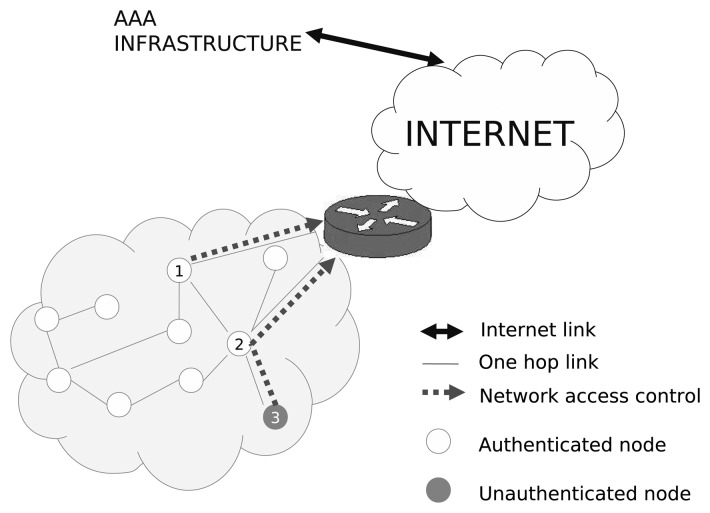
Network connectivity and access control.

In certain cases, the nodes that form these networks may require Internet connectivity through a border router (e.g., a sensor sending a measurement to a central server on the Internet), which, in turn, may need to authenticate the node to provide network connectivity. This is typically performed through an authentication process carried out using an existing authentication, authorization and accounting (AAA) server deployed in some Internet organizations. As depicted in Figure 1, node number 1 is able to send information to the Internet through the gateway, as it is an authenticated node. In the same way, this node could also send data to another authenticated node within the constrained network. In contrast, node 3 is not authenticated, and node 2 (authenticated) does not allow it to send any traffic to either the multi-hop network or the Internet.

In particular, the *Extensible Authentication Protocol* (EAP) [4] is widely used to provide flexible authentication involving AAA infrastructures. With the use of EAP and AAA and thanks to some initial pre-established credentials, a successful authentication and authorization process can provide cryptographic material and configuration parameters to different network layers with a single authentication. This enables secure access to the Internet. This general process is typically known as *bootstrapping*. However, this aspect has been an open issue until now for multi-hop networks, mainly due to a lack of a network access authentication protocol that operates at any link layer of multi-hop networks and supports AAA inter-working.

To carry out this type of operation, it is recommended to use a protocol that operates on top of IP to transport EAP between a node and the border router through several relay nodes (hops). There are two standardized protocols to transport EAP in these conditions: the *Protocol for Carrying Authentication for Network Access* (PANA) [5] and *Internet Key Exchange v2* (IKEv2) [6]. As analyzed in [7], PANA represents a lighter option to transport EAP, which is an important feature, considering the constrained resources of theses small devices. Furthermore, PANA has been designed to perform network access control, while the purpose of IKEv2 is to establish IPSec security associations. Indeed, PANA has been chosen as the protocol to carry out network access authentication and is being adopted by ZigBee IP [8] and European Telecommunications Standards Institute (ETSI) Machine-to-Machine (M2M) [9].

In this paper, we present, to the best of our knowledge, the first attempt to analyze and explore the usage of PANA in real constrained devices (*i.e.*, Internet of Things (IoT) devices). To perform this analysis, we first present the details of PANA and EAP and provide plausible simplifications that have steered the lightweight implementations of both protocols, named PANATIKI [10]. As we will analyze, these simplifications still comply with the important parts of both standards to facilitate a real deployment. We also discuss a small testbed, which we have deployed to obtain processing times and message sizes that lead to important conclusions about the usage of PANA in networks of constrained devices. To extend the analysis, we have used Cooja [11] to run simulations with several nodes.

The remainder of the article is organized as follows. Section 2 presents some related work, and Section 3 presents some important background for understanding our implementation and the corresponding results. In particular, PANA and EAP are described, as well as the most relevant aspects of the protocol, such as the associated architecture. Section 4 identifies important design decisions, which we have taken to adapt PANA and EAP to constrained devices without greatly affecting the standards. Section 5 provides some results obtained from a testbed especially designed to evaluate our implementation. Finally, we provide some conclusions and future work guidelines in Section 6.

## 2. Related Work

The network access control and bootstrapping procedures in constrained devices are important topics nowadays. The authors in [12] expose the main features of IP-based security protocols for bootstrapping. It is shown that, in general, security protocols used today on the Internet were initially designed for nodes with high computational capabilities and permanent power supply, a large amount of memory and network links with sufficient bandwidth. However, this is not the case in constrained devices. The capabilities of these devices are much lower than the general purpose ones. Furthermore, the programming paradigm and mode of operation of this type of network change.

In [13], the authors give a complete overview of the security bootstrapping solutions for constrained devices. Five areas of bootstrapping are defined: user interface, bootstrap profile, security method, bootstrap protocol and communication channel. The user interface provides the interaction between the user and the bootstrap protocol. The user interface will vary depending on the capabilities on the node. In most cases, the user interface does not exist, and all the parameters needed by the bootstrap protocol are configured statically. Those parameters are saved in the bootstrap profile, which defines what information should be exchanged during the bootstrapping process. Potentially, a single node may run the protocol multiple times with different profiles, although they should be previously defined. The security method defines supported mechanisms for bootstrapping.

The bootstrap protocol, which is the main focus of our contribution, is defined as the mean to carry out message exchanges performed during the bootstrapping process. Concretely, in [13], three possibilities are indicated as the most suitable: 802.1X [14], *Host Identity Protocol - Diet Exchange* (HIP-DEX) [15] and PANA [5]. Finally, the main aspects of each protocol are explained. Of these options, PANA is the only protocol that is able to operate between several IP hops and to interact with AAA infrastructures for network access control. In this sense, standardization bodies working on constrained devices, like the Zigbee Alliance [8] or ETSI Machine-to-Machine (M2M) [9], have adopted PANA as the bootstrapping protocol used in constrained environments. In fact, it is expected that most of the upcoming Zigbee devices will bring a PANA implementation.

In [16], there is theoretical work about the use of transport layer security-pre-shared symmetric key (TLS-PSK) for constrained devices. However, this work only reports theoretical results. No implementation has been carried out, and no practical results are shown. Moreover, there is no specific proposal about a solution for the network access control for constrained environments, just a survey about how to create cryptographic material between two constrained devices.

In [17,18], the authors propose two different solutions for network access control and key management for constrained networks. These proposals enable secure communication between constrained devices within a local network. However, this work does not address the authentication of nodes willing to exchange information on the Internet. Thus, the results obtained in these related works do not cover the problem that we try to solve in this paper.

Focusing on the available open source implementations, there are only a few PANA open source initiatives: OpenDIAMETER [19], CPANA [20] and OpenPANA [21]. OpenDIAMETER is an old implementation that no longer enjoys support. CPANA is a recent implementation based on the C language and more lightweight than OpenDIAMETER, though it is not frequently updated. Moreover, it does not include any AAA client for interacting with AAA infrastructures. Finally, OpenPANA (developed by us) is also based on the C language and includes complete support with *Remote Authentication Dial-In User Service* (RADIUS)-based AAA infrastructures. Furthermore, it supports a wide number of EAP methods. Moreover, it provides the first open source implementation of the entity, PANA Relay (PRE), which is important in constrained networks, as we will analyze in the following section. In general, these implementations have been developed for general purpose computers, and thus, they are not directly suitable for constrained devices. This motivates the development of new PANA and EAP implementations adapted to these devices, as explained herein.

## 3. Background

In the following, we provide a basic background to EAP and PANA in order to understand the rest of the paper.

### 3.1. Extensible Authentication Protocol (EAP)

The *Extensible Authentication Protocol* (EAP) [4] is a lock-step request/response protocol, which supports only a single packet (request or response) in flight. Therefore, each request message (*EAP Request*) is answered with a response (*EAP Response*). EAP allows different types of authentication mechanisms, the so-called *EAP methods* (e.g., based on symmetric keys, digital certificates, *etc.*). EAP-PSK [22] is an example of the EAP method. It assumes pre-shared symmetric key (PSK) between the EAP peer and EAP server and provides a lightweight authentication mechanism consisting of only four messages. No other secure EAP method uses fewer messages for authentication purposes. This means it is a potential candidate in constrained environments, such as IoT networks. In fact, this is the EAP method we have used for our experiments.

Every EAP method is run between an EAP peer and an EAP server through an EAP authenticator. From a security standpoint, the EAP authenticator does not take part in the mutual authentication process, but acts as a mere EAP packet forwarder.

To carry out an EAP authentication, the EAP authenticator usually starts the process by requesting the EAP peer's identity through an *EAP Request/Identity* message. The EAP peer answers with an *EAP Response/Identity* with its identity. With this information, the EAP server will select the EAP method to be performed. The EAP method execution involves several EAP Request and Response exchanges between the EAP server and the EAP peer.

**Figure 2.**
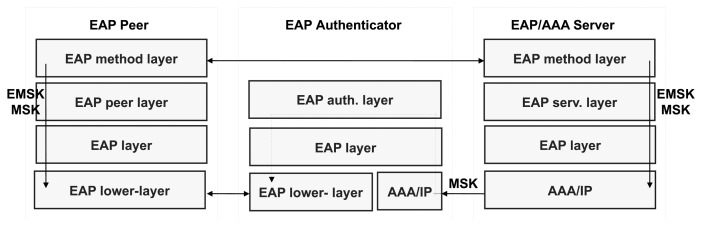
Extensible Authentication Protocol (EAP) pass-through authentication model.

Figure 2 represents the *pass-through* authentication model, which is the most deployed configuration. In this model, the EAP server and the EAP authenticator are implemented in separate nodes. Specifically, the EAP server is placed on an AAA server deployed by the network operator somewhere on the Internet. Here, communication between the EAP server and the pass-through EAP authenticator is performed using an AAA protocol, such as RADIUS [23] or Diameter [24]. In both cases, a protocol referred to as the *EAP lower-layer* protocol is used to transport the EAP packets between the EAP peer and EAP authenticator.

Certain EAP methods [25] are able to generate keying material. In particular, according to the *EAP Key Management Framework* [26], two keys are exported after a successful EAP authentication: the *Master Session Key* (MSK) and the *Extended Master Session Key* (EMSK). The former is traditionally sent to the authenticator to establish a security association with the EAP peer, while the latter must not be provided to any other entity outside the EAP server and peer. Thus, both entities may use the key material for further key derivation and, therefore, for bootstrapping purposes.

### 3.2. PANA

**Figure 3.**
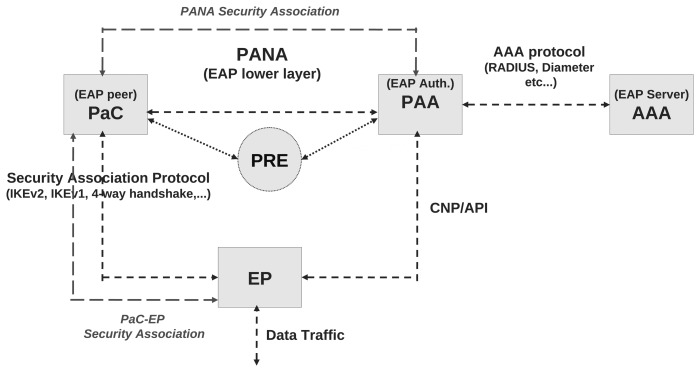
Protocol for Carrying Authentication for Network Access (PANA) Framework.

PANA [5] is an application protocol using the User Datagram Protocol (UDP) as the transport, which has been specially conceived of by the *Internet Engineering Task Force* (IETF) to carry EAP to support different authentication mechanisms for network access. Thus, it is an EAP lower-layer protocol, which is independent of the underlying network access technology. Consequently, the PANA architecture described in [27] defines several logical entities that have a correspondence with the EAP entities shown in Figure 2. This relationship is depicted in Figure 3.

The PANA network access control model considers a *PANA Client* (PaC), which requests access to the network service offered by an *Enforcement Point* (EP), such as an access point or a router. The EP is controlled by a *PANA Authentication Agent* (PAA), which is responsible for authenticating and authorizing the PaCs for the network service. The PAA communicates with the *AAA Server*, to verify the credentials provided by a PaC. If the AAA server correctly verifies the credentials, it sends authorization parameters (cryptographic material, network access lifetime, quality of service (QoS) filters, *etc.*) to the PAA. Then, the PAA transfers some configuration information to the EP by using either an Application Program Interface (API) or a Configuration Network Protocol (CNP), like Simple Network Management Protocol (SNMP) [28]. According to this operation, as depicted in Figure 3, the PaC, PAA and AAA implement the EAP peer, EAP authenticator and EAP server functionalities, respectively.

It is worth noting that a new, but optional, entity, named *PANA Relay* (PRE), has been defined in [29]. This entity is located between a PaC and a PAA and is responsible for relaying the PANA messages. From the PaC's perspective, the PRE appears as the PAA. The deployment of this entity may not be required in general scenarios, but it is essential in scenarios where the PaC cannot make contact directly with the PAA. This situation is typical in IoT environments, like that shown in Figure 1, where the unauthenticated node can only interact with the nearest authenticated node, but not with an entity several hops away (*i.e.*, PAA).

The PANA operation is performed along four phases. First, the *authentication and authorization phase* is initiated by the PaC through a *PANA-Client-Initiation* (PCI) sent to the PAA. In this phase, the PaC and the PAA exchange several *PANA-Auth-Request/Answer* (PAR/PAN) messages, which are used to negotiate some parameters, such as the integrity algorithms used to protect PANA messages. They also exchange PANA messages transporting EAP to perform the authentication and to establish a so-called *PANA session*. At the end of this phase, two security associations are established. On the one hand, the *PANA Security Association (SA)* is established between the PaC and the PAA in order to protect the integrity of the PANA messages. To build this security association, both PaC and PAA derive the *PANA_AUTH_KEY* from the MSK obtained once the PaC has been successfully authenticated with EAP. This *PANA_AUTH_KEY* is the one used for integrity protecting the PANA messages. On the other hand, after the PANA SA has been set, a *PaC-EP SA* may be established to protect data traffic between the PaC and the EP

Once the PaC is successfully authenticated, the protocol enters the *access phase* in which the PaC can use the network service just by sending/receiving data traffic through the EP. If the session is about to expire, typically a *re-authentication phase* occurs to renew this session lifetime, as well as the associated security associations. The PaC or PAA can terminate the session (e.g., the PaC wishes to log out the network access session) during *termination phase*, where resources allocated by the EP for the PaC are also removed.

If the PANA authentication involves a PRE between the PaC and PAA, the PRE encapsulates each message sent by the PaC in a new message, named PANA-Relay (PRY), and sends it to the PAA. The PAA encapsulates its answer in another PRY message and sends it to the PRE. Finally, the PRE decapsulates the message sent by the PAA and forwards it without modification to the PaC.

## 4. Adapting PANA and EAP for Constrained Devices

Resources, such as storage, computation or networking capabilities, that we find in constrained devices are much smaller than in general purpose computers. In fact, constrained devices can be classified according to the available read-only memory (ROM) and random access memory (RAM) [30]. Using this classification, we focus our efforts on constrained devices belonging to class 1 and class 2, given that they are the only ones that have enough power to run a protocol stack specifically defined for constrained devices. While class 1 devices present an available RAM of approximately 10 kB and a supported ROM size of approximately 100 kB, class 2 devices are more powerful, having approximately 50 kB of RAM and 250 kB of ROM available. This circumstance motivates, before starting to implement PANA and EAP, the analysis of a set of key aspects in the design of these protocols. The goal is to determine if certain simplifications are possible without breaking the standards.

### 4.1. Preliminary Considerations

**Figure 4.**
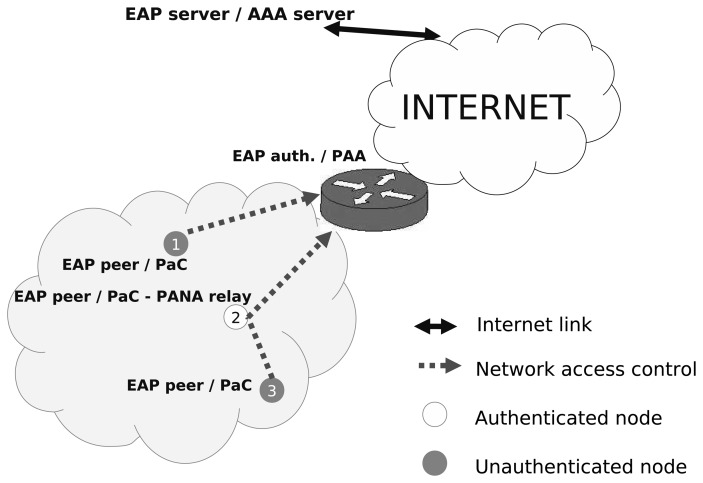
PANA framework for network connectivity and access control.

To perform an authentication, an *unauthenticated node* will have to implement the PaC with the EAP peer functionality and the client part of the EAP methods. Once this node is authenticated, it becomes an *authenticated node* and may act as a PANA relay to forward PANA messages between a new unauthenticated PaC and the PAA deployed. Therefore, a node joining the multi-hop network and trying to send traffic to the Internet will have to implement both PaC and PRE functionality. Thus, these implementations must be optimized to preserve resources in the constrained device. In contrast, the PAA will be typically co-located with the border router, which is assumed to have enough resources to run a general PANA implementation. This mapping is represented in Figure 4.

Our first attempt to deploy PANA in constrained devices was to adapt OpenPANA to the requirements imposed by these constrained devices. However, the source code of OpenPANA is not well adapted for constrained environments. For example, it makes use of heavy libraries for constrained devices, like open secure sockets layer (OpenSSL) [31] or implements multiple EAP methods to provide flexibility. However, this variety increases the source code size. This has led us to implement, from scratch, a PaC and a PRE specially adapted for constrained devices. This implementation is based on the Contiki OS, which is the operating system used by the devices we have used for our real experience.

For these implementations, we have followed documents *Request For Comments* (RFC) 5609 [32], RFC 4137 [33] and RFC 6345 [29], which describe the PANA state machine, the EAP state machine and the PRE behavior, respectively. Nevertheless, it is convenient to analyze how these state machines can be simplified without removing any mandatory behavior of the standard protocols.

Additionally, it is important to make use of the hardware resources that these devices already offer. This means adapting the variables' length to the word length of the microprocessor or using available hardware cryptographic modules. For example, several constrained devices already integrate certain hardware implementations of basic cryptographic functions. PANA and EAP typically require a certain cryptographic suite to perform authentication and key management. In PANA authentication, the establishment of the PANA Security Association (SA) requires cryptographic operations apart from those required by the chosen EAP method. Thus, an important design decision is not only to keep the same cryptographic suite in both PANA and the EAP method so that it can be reused, but also to try to select this cryptographic suite in such a manner that most of the required cryptographic functions are already implemented by the device's hardware.

In addition, we need to reduce the number of messages, variables, features, *etc.*, as much as possible. Each non-mandatory feature and byte we can save will be key in achieving a reduction in other parts. As a design principle, this must be done following the standard's rules in order to achieve interoperability with other implementations.

Below, we focus on specific viable simplifications in the state machines of the PaC, EAP peer and the EAP method layer of the EAP peer. It is worth noting that the EAP method layer is implemented by the EAP method, so it will depend on the EAP method selected. For the purpose of our study, we have chosen the lightweight EAP-PSK (pre-shared key) [22]. This election is not random. Apart from being based on symmetric cryptography (lighter than asymmetric one), the cryptographic suite used by EAP-PSK uses the advance encryption standard (AES) as the main algorithm for its operation. This algorithm is implemented in the hardware in many constrained devices.

Given that the PANA Relay entity is also implemented in this work, we also provide some insights into its implementation. Finally, although PAA is not installed in constrained devices (we use the PAA distributed in OpenPANA), some specific behavior of the PAA is expected to handle PANA authentications coming from a constrained device. We discuss the details of this behavior.

### 4.2. EAP Peer State Machine

In general, the EAP peer state machine definition is simple (see Appendix); however, some simplifications are still possible. In particular, we can safely obviate the *portEnabled* variable and the *Disabled* state, because they were inherited from legacy technology (e.g., 802.1X technology), and we can assume that *portEnabled* is always TRUE and *Disabled* is FALSE.

**Figure B1:**
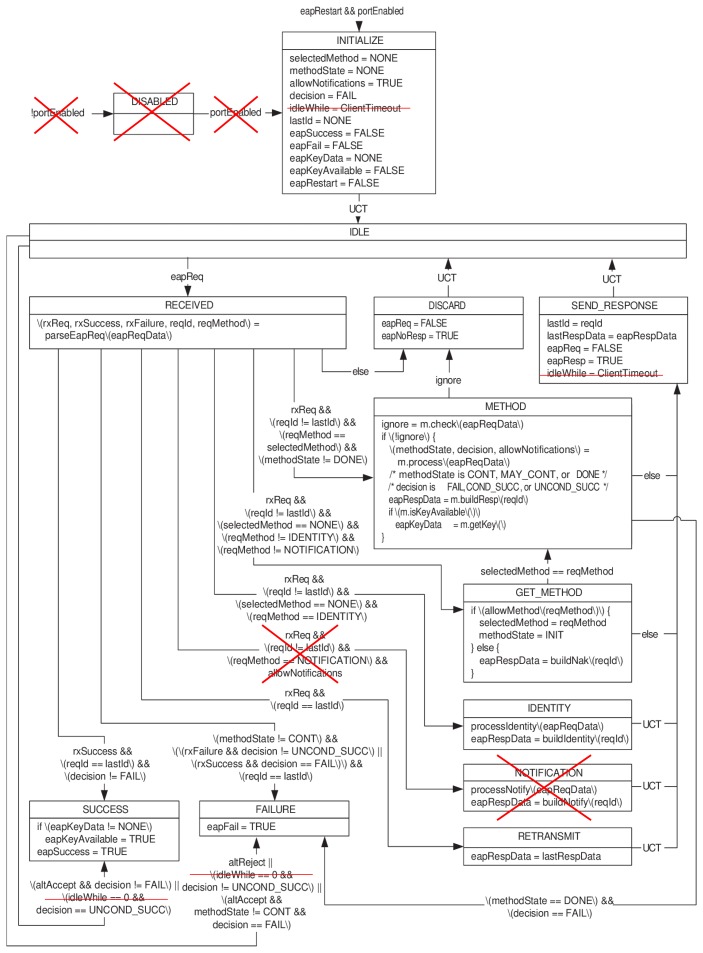
Light-weight EAP peer state machine.

We have also deleted the timeout management in the EAP peer. This timeout is established by the peer, so that it avoids waiting for a EAP Request indefinitely. Nevertheless, this situation can also be discovered if the EAP lower layer notifies the EAP peer that the authenticator has not sent any message after a certain period of time. Since PANA provides this functionality, it is not worth duplicating functionality in both layers. This simplifies some transitions and avoids extra verification and management of this timeout (see Figure B1).

Finally, we have deleted the *Notification* transition. A *Notification* message is *optionally* used to transport a displayable message from the authenticator to the peer. However, it is not widely used, and certain EAP methods prohibit or do not recommend its usage, because this message is not securely protected. EAP-PSK is an example. Thus, our EAP peer state machine removes this state and its management, since it is not essential.

### 4.3. PANA Client State Machine

In general, the complete PaC state machine can be simplified to fit better in constrained devices. Therefore, we need to make some decisions to get a lighter version without affecting the mandatory parts of the standard.

**Figure 5.**
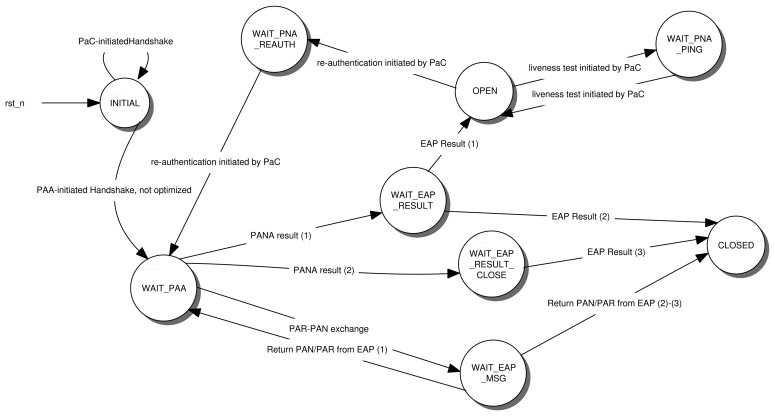
PANA light-weight state machine.

Some hints of this simplification process can be found in [7], where we have some useful guidance and recommendations about how the PANA client can be tailored to meet the requirements for constrained environments. From this contribution and our own experience, we can establish a set of simplifications of the PaC state machine. Figure 5 shows a simplified PaC state machine after the following simplifications.

#### 4.3.1. PANA Session Initiation Phase

There are two ways of initiating a PANA (re-)authentication: *PaC-inititated* or *PAA-initiated*. Taking into account that constrained devices usually have a dormant mode to save energy power, a PAA-initiated authentication may fail if the device is sleeping. Thus, a PaC-initiated authentication is recommended, so that the PaC can start the authentication (or re-authentication) once it is awake. This eliminates the states related with the processing of the PAA-initiated authentication (see Table A1 in Appendix).

The recommendation of supporting only PaC-initiated (re-)authentication applies not only to sensors generating upstream traffic, but also to actuators, which additionally support the reception of downstream traffic. It is worth noting that the authentication process is independent of the specific application run by the sensor or actuator. Furthermore, authentication is only needed once, before the specific application messages are exchanged. Thus, when an actuator is installed (or when it is ready to send/receive specific application information), it can perform the PANA authentication by triggering the PaC-initiated mechanism. Once the actuator is authenticated, it is ready to send/receive any specific application message.

#### 4.3.2. Piggybacking EAP Message

EAP messages may be transported in *PANA-Auth-Request* (PAR) and *PANA-Auth-Answer* (PAN). By default, EAP messages are transported only by PAR messages within an EAP-Payload Attribute-Value Pair (AVP), while PAN is used just to acknowledge receipt of a PAR. Thus, PAA sends PAR messages with an EAP Request, while PaC with an EAP Response. However, this implies extra signaling and overload, since PAN transports just a confirmation. Nevertheless, PANA does allow the *EAP piggyback* mechanism, which consists of transporting EAP messages in both PAR and PAN messages instead of sending a PAN just as an acknowledgment of the receipt of a message. In this case, the PAN is also used to transport an EAP message, and therefore, this mode considerably reduces the number of messages exchanged in the authentication process. Nevertheless, it also has other implications. Due to EAP piggybacking being always in use in our deployment to save resources, the transitions and states defined in the PANA state machine that support non-EAP piggybacking can be avoided (see Table A1.{5,6,7} in Appendix). This considerably reduces the complexity of the state machine.

In addition, the PaC would only receive PAR messages, and it would only send PAN messages. This feature has two implications. One is that the transitions that check if a PAN message has been received can also be deleted (see Table A1.{6}, in Appendix). The second implication is analyzed below.

#### 4.3.3. PaC Retransmissions Avoided

Assuming the use of EAP piggybacking, our PaC will only send PAN messages. In PANA protocol, only PAR messages can be retransmitted. In other words, we can simplify the PaC state machine by reducing the operation in the states that pay attention to the retransmission mechanism (see Table A1.{1,2}, in Appendix). Indeed, the only task that PaC has to do is to answer with the same PAN upon receiving a duplicated/retransmitted PAR.

#### 4.3.4. PANA Re-Authentication Phase and PANA Ping Message

By sending PANA-Notification-Request/Answer (PNR-PNA), PANA can initiate a keep-alive mechanism or initiate the re-authentication. In the standard, this exchange can be initiated by either the PaC or the PAA. However, in constrained environments, the PaC may be sleeping and would not answer with any PNA if the PAA decides to send a PNR to verify whether the PaC is alive or start the re-authentication [7]. This could lead the PAA to think that the PaC is not operating anymore and to terminate the PANA session as a consequence. It is therefore safer to assume that PNR will always be sent by the PaC if it needs to test the availability of the PAA or to perform a re-authentication. For example, the PaC could send a PNR to the PAA every time the device wakes up. Clearly, this allows the PAA to detect that PaC is alive.

Thus, under this assumption, we do not need to implement in the PaC state machine the processing of any PNR coming from the PAA (see Table A1.{3,4,8,10}, in Appendix). Furthermore, the preparation and construction of the the corresponding answer PNA can be obviated.

#### 4.3.5. Session Termination Phase

To terminate a PANA session and release any associated resource, PAA or PaC can send a *PANA-Termination-Request* (PTR), which will be answered with a *PANA-Termination-Answer* (PTA). Moreover, if the PAA sends a PTR to a dormant node, this will not answer. Another way to release these resources is to wait until the PANA session lifetime expires. This does not require any message exchange and processing. Therefore, avoiding extra states or transitions in the PaC state machine (see Table A1.{8,9,10,11}, in Appendix).

This has the drawback that PAA will have to keep the state during the whole lifetime of the PANA session. Nevertheless, we can assume that PAA will be placed on a device (e.g., border router) with enough resources to keep this state safely. Below red part is the footnote to the main text.

### 4.4. PANA Security Association

Another key part that can be optimized is the PANA Security Association (PANA SA) and the message integrity protection once the authentication has successfully been completed. In particular, a PANA SA is established thanks to the derivation of a key named *PANA_AUTH_KEY*. This key is obtained from the MSK exported by the EAP method by applying a key derivation function named PRF+ (pseudo random function) defined in [6]. The *pseudo random function* (PRF) used to implement PRF+ is based on the use of *hash-based message authentication code - secure hash algorithm 1* (HMAC-SHA1). This is a keyed-hash function that provided us with the integrity protection of the messages. Once the authentication has successfully been performed and the PANA_AUTH_KEY has been generated using the mechanism shown above, the integrity protection of the messages (PANA_PDU) is achieved by including an authentication tag contained in the AUTH AVP.

However, HMAC_SHA1 is not implemented in the hardware we use for our experimental testbed. Instead, the device implements an AES module, which is a common algorithm included in cryptographic processors of constrained devices. Taking into account the principle of taking as much advantage as possible of the hardware resources, we have replaced HMAC_SHA1 for the *advanced encryption standard - cipher-based message authentication code* (AES-CMAC) algorithm [34] to derive the PANA_AUTH_KEY and to provide integrity to PANA messages. It is worth noting that, although PANA defines HMAC_SHA1 as the mandatory cryptographic suite to be supported, other cryptographic suites may be used optionally (e.g., AES). In order to get our proposal working, we demand that the PaC supports only AES-CMAC, while the PAA facing a constrained network must support both HMAC_SHA1 and AES-CMAC. In this way, the solution is scalable. The PAA can handle the authentication of different devices (The PAA will use HMAC_SHA1 as the cryptographic suite for authenticating a general purpose device, while AES-CMAC will be used for authenticating a constrained device), while still following the PANA specification. We affirm that the inclusion of both AES-CMAC and HMAC_SHA1 in the PAA is feasible, as we have already done it in our project, OpenPANA [21].

In addition, as we will see in Section 4.5, the EAP method we have selected in our experimental testbed is EAP-PSK, which also uses AES as the main cryptographic algorithm. In this manner, we still keep the same cryptographic suite in every part of the protocol stack. Therefore, AES is not only used in PANA_AUTH_KEY generation and AUTH AVP generation, but also in the EAP method. As is evident, we recommend that other developers change this function and use another cryptographic algorithm, if the device employed supports other types of functions, in order to follow the same principle.

Nevertheless, the choice of AES-CMAC as the cryptographic algorithm for integrity has some implications. Specifically, it implies that the PANA_AUTH_KEY must be 16 bytes in length (the HMAC_SHA1 result is a 20-byte length). This is not a problem, because applying AES-CMAC to derive PANA_AUTH_KEY already generates a 16-byte output with a single iteration. However, the MSK used as a parameter of AES-CMAC is 64 bytes in length. AES-CMAC only accepts 16-byte length keys as the parameter. The solution that we have adopted is the use of the 16 most significant bytes of this MSK.

### 4.5. EAP Method: EAP-PSK

The possibility of choosing between several EAP methods is one of the advantages of using EAP. However, several choices imply adding more source code to the constrained device. Thus, it is convenient to select only one EAP method and implement it. In particular, as pointed out in [7], an EAP method that minimizes the number of messages is preferable. This is the case of *Extensible Authentication Protocol - Generalized Pre-Shared Key* (EAP-GPSK) [35] or EAP-PSK [22], which have the minimum number of messages for an EAP method. Moreover, they are based on symmetric cryptography, which is better in terms of resource consumption than other methods based on asymmetric cryptography, such as EAP-TLS [36].

Between EAP-GPSK and EAP-PSK, we have used the latter, because, apart from being very similar to EAP-GPSK, it relies on a single cryptographic primitive AES-128, which lets us keep the same cryptographic suite in all our protocol stacks. Besides, EAP-PSK is supposed to be lightweight and well suited for any type of device, especially those of small processing power and memory. Finally, for simplicity, EAP-PSK has also chosen a fixed message format, which eases the processing.

EAP-PSK defines two modes of authentication: *EAP-PSK Standard Authentication* and *EAP-PSK Extended Authentication*. We have used the *Standard Authentication* mode, which builds a protected channel with less source code and complexity. It is worth noting that this protected channel contains a value in the last message named P_CHANNEL_1, which is cryptographically verified by the EAP peer part of EAP-PSK. However, the same message contains a Message Authentication Code (MAC). We argue that the MAC verification is enough for the EAP peer to authenticate the EAP server, so verifying P_CHANNEL_1 is redundant. Thus, we have removed the source code to verify P_CHANNEL_1 in the constrained device.

Finally, the use of EAP-PSK implies that a PSK key must be distributed in advance to the device to be authenticated. Given the resource constraints in sensor devices, our proposal is based on the assumption that a device has been manually pre-configured with a PSK that is known *a priori* to the authentication server. This assumption is reasonable since a PSK can be included in the manufacturing process of a device and stored in the authentication server in charge of authenticating the corresponding sensor.

### 4.6. PANA Relay

The PANA Relay entity (PRE) is located between the PaC and the PAA, and its main function is to forward PANA messages from the PaC to the PAA and *vice versa*, without keeping any session state. Thus, the complexity associated with this entity is relatively small.

According to RFC 6345 [29], the functionalities to be implemented are mainly: reception of messages from different sources (*i.e.*, PaC and PAA); encapsulation of PANA messages sent by PaC to the PAA in PANA-Relay (PRY) messages; and the corresponding decapsulation of the answer sent by the PAA to the PaC, also contained in a PRY message.

### 4.7. PANA Agent

While the PANA Agent (PAA) does not require any particular simplification (we assume that it would be placed in the border router or any other device with suitable resources), it is important that PAA establish a set of policies that control its behavior when it is controlling a network with constrained PaCs. These policies are related to the PaC's behavior that we have described in previous sections, namely:

- PAA should not initiate (re-)authentication, even if the session lifetime is about to expire. Instead, it must wait for a notification message from the PaC indicating the initiation of a (re-)authentication process.
- The EAP piggyback option will be used. Given that lightweight PaC assumes its usage, this option is activated in the PAA, as well.
- The PAA will only answer PNR messages and will not send PNR to the PaC. (*i.e.*, such as PANA Ping or to start a (re-)authentication process). Similarly, PAA should avoid sending a PTR message to terminate a session.
- The PANA SA must be only removed when the associated lifetime expires. We assume that the PaC is in charge of sending a PANA notification message before lifetime expiration to perform the re-authentication.
- The AES-CMAC function must be available as a PRF+ and integrity function, since the PaC deployed in an IoT device will support only this cryptographic suite. Thus, the same function must be supported in the PAA.

## 5. Evaluation and Result Analysis

In this section, we show some evaluation results that we have obtained from our implementation and the hardware platform used.

### 5.1. Deployed Testbed

First of all, it is necessary to describe the testbed used in this evaluation process. We have used the JN5139 hardware [37] as an example of a constrained device. The JN5139 module has only 96 kB of RAM and 128 kB of ROM. These limitations have been taken into account, since we must adjust our implementation to fit in this space. It has a wireless communication module based on IEEE 802.15.4 [2] and implements a 128-bit AES encryption module, which allows faster encryption operations. As the operating system, it uses Contiki OS [11], which is specially adapted to this type of constrained device. Programming in Contiki OS is based on the *american national standards institute* (ANSI) C language.

**Figure 6.**
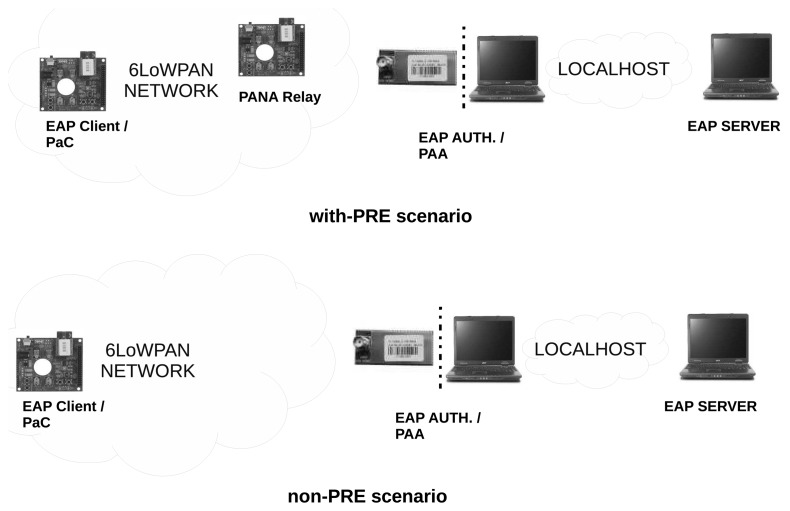
Evaluation testbed.

The entities that take part in the testbed are described in Figure 6. In particular, two scenarios have been deployed. On the one hand, the *non-PRE* scenario, in which the PaC is deployed in a JN5139 module [37] that connects directly with a bridge 6LoWPAN-Ethernet.

Conversely, the PAA is placed on a regular PC, which is connected to the Ethernet network. Thus, once a PANA message reaches the bridge, this hardware sends the message to the computer where the PAA is running. The same process is done on the way back. On the other hand, we have the *with-PRE* scenario, where, unlike the non-PRE one, the PaC is not directly connected with the PAA through the bridge, but by means of a node acting as the PRE. Thus, the messages created by the PaC are sent to the PRE, and this entity will forward the message to the PAA using the bridge over the 6LoWPAN network. Once a message reaches the PAA, this entity will send the answer to the PRE, which will forward it to the PaC.

Finally, the server part of EAP-PSK is already implemented by the RADIUS server. The RADIUS messages are sent over the localhost interface, since the RADIUS server is located in the same computer as the PAA. While the PAA has been deployed by using OpenPANA [21] (version 0.2.3), the RADIUS server implementation is based on FreeRADIUS [38] (version 2.0.2). Both the PAA and RADIUS server have been deployed over an Ubuntu 12.04 32-bit operating system. Elsewhere, PANATIKI [10] (version 0.1) has been used for PaC and PRE implementations.

The *non-PRE* and *with-PRE* scenarios have been also simulated on Cooja [39] to experiment with more nodes. Cooja is a simulator of constrained networks available in the Contiki OS software. It allows the simulation of constrained nodes running a Contiki OS-based user application suitable for the inherent constraints to such nodes. In particular, *PaC*, *PRE* and *6LoWPAN Ethernet bridge* functionalities have been loaded into simulated z1 nodes [40], which are more constrained than JN5139 devices. In fact, they have 92 kB of ROM and only 8 KB of RAM. The rest of the elements of our testbed (*i.e.*, PAA and the EAP server) have been deployed in a real computer, as explained for the real hardware-based deployments. Given that simulated nodes (*i.e.*, Z1 nodes) have different capabilities than real hardware nodes (*i.e.*, Jennic nodes), a small set of changes has been needed in both the OpenPANA and PANATIKI software. In particular, OpenPANA (version 0.2.4) has been used to run PAA, while PANATIKI (version 0.2) has been used for PaC and PRE simulated entities. RADIUS server software remains as the same version as in the hardware-based deployment. Finally, the software needed for the simulated Ethernet bridge has been adopted by the examples available in Contiki release 2.6.

These Cooja-based simulations allow us to test our deployment according to variables that we could not check with the real hardware-based testbed. In particular, we have tested our implementation depending on a variable number of PaCs and a variable packet loss ratio.

### 5.2. Results

We have carried out two deployments of our testbed. The first deployment has been based on real hardware, while the second deployment has been simulated on Cooja with additional nodes. In this section, we present two sets of results corresponding to each deployment.

#### 5.2.1. Results with Real Hardware

The three main aspects that we have analyzed in our real hardware-based testbed are memory occupied for our implementation, message length and execution time, which are vital in the deployment of protocols in constrained devices. Indeed, obtaining a small as possible source code is essential to fit the results into the constrained devices. Moreover, the length of the messages exchanged is crucial in order to reduce the exchange time and to avoid undesirable fragmentation over the IEEE 802.15.4 link. In fact, the maximum message length allowed without fragmentation in 6LoWPAN is currently 127 bytes. Therefore, bearing in mind that some of these bytes are used in the network and transport layer headers, the PANA messages should be small enough in order to fit this restricted length. Thus, short messages will be more suitable in these constrained environments. Finally, we will also study the execution times, taking into account the constrained capabilities of the microprocessor.

##### Memory size

Table 1 shows the memory size used by our implementation. We have used the following method to collect these values. First, we take the measurement of an empty main function. This measurement provides us with a reference value, which includes the size of a basic Contiki OS installation.

From that point, we have added a functionality to measure the increment in memory size. For example, if we add the PANA state machine definition (*i.e.*, transition table, associated functions, *etc.*) to the empty main function and, then, we take a new measurement, the difference between the new measurement and our reference will give us an approximate memory size used by the PANA state machine definition. By repeating the same process for each module, we obtained the results shown in Table 1.

The value of these measurements can be obtained from two files after compiling the application: *application.jndevkit.hex* and *application.jndevkit*. The first is directly installed in the JN5139. The second is used with the tool, *ba-elf-size*, to obtain the code size shown in Table 1. The tool shows three main segments: the code segment (.text), the data segment (.data) and the uninitialized variables segment (.bss). The two columns represent how much memory is used in both ROM and RAM.

It is worth explaining some of these values. The definition and message management in the EAP state machine part have no cost in terms of RAM memory, because the values are shared with the PANA state machine and their contribution is provided in the PANA state machine part. In addition, the cryptographic suite implementation part does not consume RAM, due to, as we have noted, optimizations performed by the compiler. In fact, the cross-compiler used for Contiki OS applications based on the JN5139 platform is able to make this type of optimization to reduce the whole application size considerably.

Under these considerations, the executable file *application.jndevkit.hex* needs 77,404 bytes of ROM memory and 8,868 bytes of RAM. These values involve the whole operating system, as well as the different PANA functionalities defined in this work. Focusing only on PANA-related implementation, this only represents under 15% of the total in terms of both ROM and RAM memory.

##### Message length

Another important aspect to analyze is the length of the messages that travel over the wireless link. Table 2 shows the size of every message that takes part in the PANA authentication and re-authentication phases. It is worth noting that the table shows two *EAP Request/Response Identity* exchanges. This double *EAP Request/Response Identity* handshake is carried out in both authentication and re-authentication process. The reason is that FreeRADIUS supports an experimental EAP-PSK module that always starts with the *EAP Request/Response Identity* before starting with EAP-PSK functionality. While the first exchange is started by the PAA, the second is started by the FreeRADIUS implementation. Thus, this second exchange can be completely avoided in a more optimized implementation of this module in FreeRADIUS. However, we show it to highlight the behavior of the real implementation.

Under this consideration, a comparison between the relayed messages (with-PRE case) and non-relayed messages (non-PRE case) has been performed to get these message sizes. The Wireshark [41] tool was used. The tool was installed in the PAA entity.

From Table 2, the PANA messages with bigger sizes are the ones that carry EAP-PSK messages or the EAP-Success message. This is because these messages carry most of the authentication information. Due to these sizes and 6LoWPAN limitations in terms of maximum packet size (127 bytes), these messages will have to be fragmented in the wireless link; so, the authentication and re-authentication times will be higher. Messages expected to be fragmented have been marked with an asterisk (*).

The other messages are smaller given that they are just notifications of events, such as authentication initiation or re-authentication initiation, or they exchange small information, such as the identifier of the cryptographic suite or the client identifier itself. Thus, they are not fragmented.

Finally, as shown in Table 2, the messages in the with-PRE case are bigger than the non-PRE case. The reason is that the with-PRE case includes the PRY transport overhead, which is 52 bytes. Thus, encapsulated PANA messages may also generate more fragmentation in the scenario in which the PRE is involved. Indeed, fragmentation is the main reason for the differences between the execution times in each scenario, which we analyze below.

##### Processing time measurements

The time measurements have been taken in tick units since Contiki OS provides a generic clock interface that gives us the time in clock ticks (one system tick is equivalent to one millisecond).

**Figure 7.**
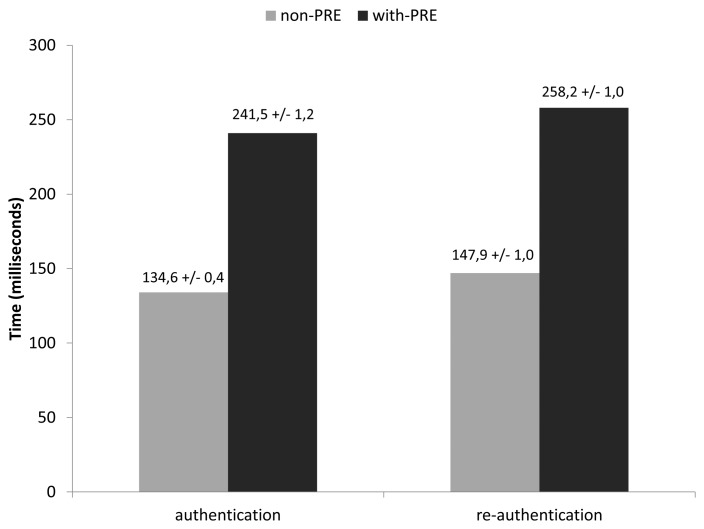
Total mean authentication and re-authentication times.

Figure 7 gives the execution time of the whole authentication and re-authentication phases in both scenarios. In general, the re-authentication phase takes longer than the authentication one. The main reason is that messages exchanged in the re-authentication are typically bigger, since they include the authentication tag (AUTH AVP). Thus, there is more fragmentation, which increases the general re-authentication time. Moreover, when the PRE entity is used, the use of the PRY message (for encapsulating PANA messages between PRE and PAA) increases the length of the messages sent over the wireless link. Thus, the fragmentation increases even more, and the final authentication and re-authentication processing time in with-PRE scenario is higher than the non-PRE scenario (apart from the fact that the with-PRE scenario includes an additional hop). Nevertheless, authentication and re-authentication times without using a PRE and with the use of a PRE can be considered reasonable compared with other studies on typical PANA authentication time in non-constrained environments [42]. This shows that PANA, with the simplifications proposed in this paper, can be used for bootstrapping network access control with AAA-inter-networking in constrained devices. Indeed, these authentication and re-authentication operations do not need to be performed frequently (*i.e.*, every 8 h, according to the guidelines given in [26]).

To observe these general times in more detail, we show the per-message mean processing time with a 95% confidence interval for authentication and re-authentication in Tables 3 and 4, respectively. The time associated with each message measures the number of milliseconds between processing a received PANA message (if any), constructing the response and sending it. From the results, we have deducted that a message that needs to be fragmented by the sender lasts ≈6 ms. For example, when PaC sends the **PAN(EAP-PSK 2)** message during the authentication phase in the non-PRE scenario, it lasts ≈3.8 ms. However, during the re-authentication, this message includes the AUTH AVP, which means fragmenting the **PAN(EAP-PSK 2)** in the PaC. Hence, we can observe that the processing time rises to ≈10.6 ms. This increment of around 6 ms is always observed when any entity needs to fragment a message that it is going to send. Messages following this hypothesis have been marked with a double asterisk (**). Regarding the reception, it is worth noting that the time shown does not include this fragmentation processing time, since we have not been able to measure it. The reason is that the receiver already obtains the complete message directly from the reception socket in an asynchronous event.

Focusing on Table 3, the processing of each message in the PaC during the authentication entity lasts almost the same time. In fact, the messages are of the same size in both the with-PRE and non-PRE scenarios. However, we can observe that this time has increased significantly in the PRE entity, especially in the encapsulated messages from PaC to PAA. The main reason is not the generation of PRY messages to encapsulate the PAN, but the processing time devoted to fragmentation procedures, as discussed above. However, when the PRE receives a PRY message from the PAA with an encapsulated PAR, which is also fragmented, the processing time does not reflect the time dedicated to reconstructing these received fragments. As discussed, the reason is that the packet is obtained from the socket that is already complete without any fragment. Thus, in general, the processing time in the PRE for the PAN messages are bigger than the PAR ones (a difference of about 6 ms).

Similarly, this can be applied for the re-authentication times shown in Table 4, but with some differences. First of all, the processing time of every message is a bit longer, given that every message has the AUTH AVP in the re-authentication phase. Thus, some additional cryptographic operations with respect to initial authentication occur. More importantly, including the AUTH AVP also increases the size of the message, and fragmentation may occur. As an example, while during the initial authentication, the PaC does not fragment the PAN messages carrying EAP-PSK 2 and EAP-PSK 4 messages, during the re-authentication and due to the inclusion of the AUTH AVP, the PaC must fragment PAN messages, and the processing time increases.

#### 5.2.2. Results with Simulations

By means of simulation in Cooja, we have taken measures of the same parameters (messages processing time, processing time per message and total execution time), but now varying the numbers of PaCs and the packet loss ratio characterizing the constrained network (in the experiments with real hardware, we only had one PaC and one PRE). In this manner, we obtain further details regarding a potential deployment with more nodes.

In real life, more than one user (*i.e.*, PaC) starts the (re-)authentication process at the same PAA to get network access. We simulate a variable number of users ranging from two to five. Communications carried out over constrained networks also have to deal with certain packet loss. Thus, we have simulated a variable packet loss ratio ranging from zero to 30% (Note that a constrained network with a packet loss ratio bigger than 30% is not considered operative any more).

##### Message processing time

**Figure 8.**
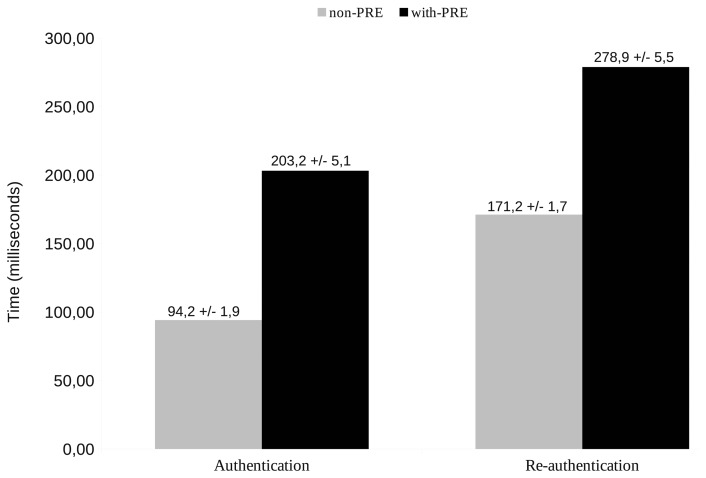
Mean message processing time (Note that in both cases, authentication and re-authentication, time(non-PRE) = messages processing time in PaC. Thus, time (with-PRE) = time(non-PRE) + messages processing time in PRE.).

The time has been measured directly from the time values provided by the Cooja simulator. When a simulation is run in Cooja, every logged action is timestamped. These timestamps have been used to calculate the processing time needed for the messages involved in the authentication and re-authentication process with a trust interval of 95%, as depicted in Figure 8. Since we are only interested in our PANATIKI implementation, the data only shows the message processing time in the PaC in the non-PRE case and the sum of PaC and PRE times in the with-PRE scenario. The transmission time is not considered in this case, because it depends on the packet loss ratio.

We have found out two main reasons to explain the different times between not only authentication and re-authentication processes, but also between *non-PRE* and *with-PRE* cases. In Cooja-based simulations, the AES-based cryptographic functions must be implemented in software. The software-based cryptographic functions take much longer to be computed than those based on real hardware. Thus, messages that need more cryptographic computation will lead to a longer processing time. This is the case of re-authentication messages, given that they all carry an AUTH AVP, which has to be cryptographically computed. In contrast, authentication messages do not have it (except for the last PAR and PAN messages). The *with-PRE* scenario implies two different computations for the same single message (*i.e.*, a single message has to be computed on PaC and relayed by the PRE). Hence, the message processing time in the *with-PRE* scenario is bigger than in the *non-PRE* scenario.

**Figure 9.**
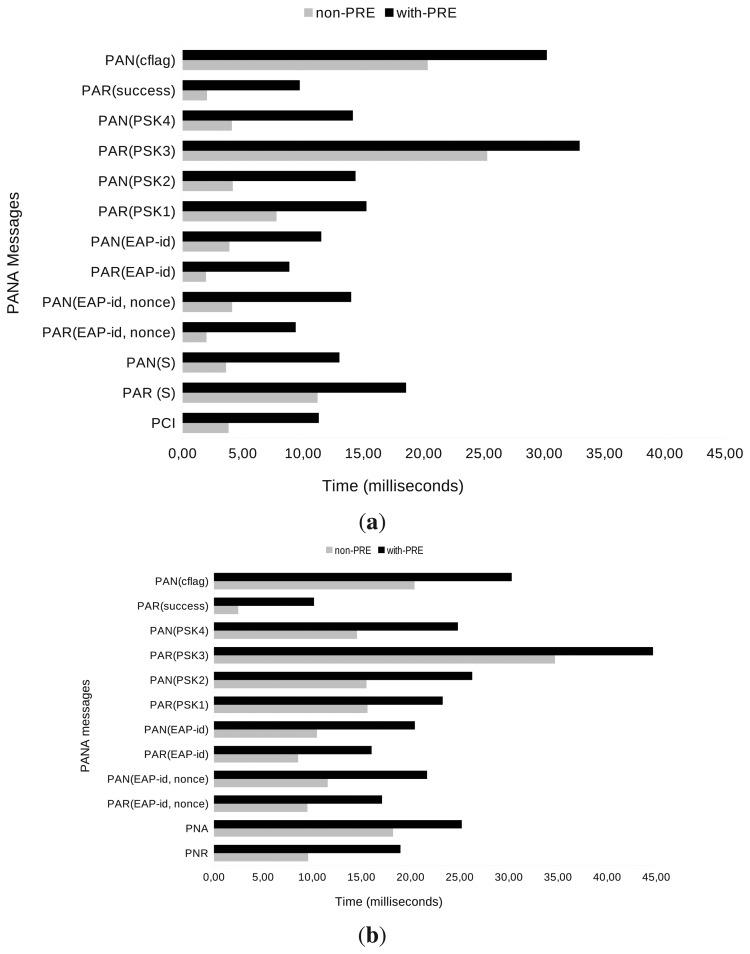
Mean processing time per message during the (re-)authentication phase (Note that in both cases, authentication and re-authentication, time(non-PRE) = messages processing time in PaC. Thus, time(with-PRE) = time(non-PRE) + messages processing time in PRE). (**a**) Mean processing time per message during the authentication phase; (**b**) mean processing time per message during the re-authentication phase.

**Figure 10.**
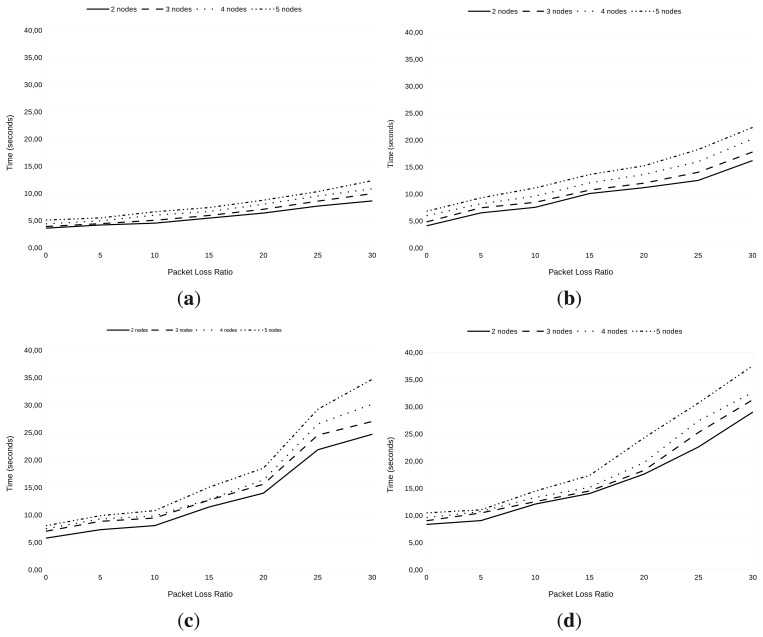
Execution times based on Cooja simulations. (**a**) Authentication execution time in the non-PRE scenario; (**b**) re-authentication execution time in the non-PRE scenario; (c) authentication execution time in the with-PRE scenario; (**d**) re-authentication execution time in the with-PRE scenario.

Figure 10a,b shows the processing time needed per message on average for the authentication and re-authentication processes. Messages are shown from bottom to top in the same order as they are transmitted. First, messages carrying EAP-PSK information created by cryptographic functions (see PAR(PSK3) in Figure 9a) and messages carrying the AUTH AVP (see PAN(cflag) in Figure 9a or every message in Figure 9b) need more processing time, because of the high computation cost of software cryptographic functions. Second, in the *with-PRE* scenario, an extra processing time is needed for each message, given that every message has to be processed by the PaC and again by the PRE in order to be relayed to the PAA.

##### Execution time measurements

We have studied the time for the whole authentication and re-authentication process depending on a variable number of nodes (*i.e.*, PaCs) and a variable packet loss ratio. We have also set up the different nodes in such a way that they are sequentially (re-)authenticated. The results appear in Figure 10.

From Figure 10, we can deduce that: (1) the re-authentication process takes longer than the authentication process (this is in line with to the reasoning stated above on this document); (2) the bigger the number of nodes being (re-)authenticated, the longer the time needed to authenticate each one, on average; (3) by increasing the packet loss ratio held in the constrained network, the (re-)authentication time is also increased.

Furthermore, in the *with-PRE* case (see Figure 11c,d), the (re-)authentication time is longer than in the *non-PRE* case (see Figure 11c,d). This is due to the longer message processing time (as explained in Figure 8) and to the existence of an extra link; so, an extra transmission is needed to successfully complete a (re-)authentication process.

Finally, we have observed in the Cooja-based tests a variability of the results obtained for (re-)authentication times in every case tested. However, we have presented a relatively small standard deviation of the results concerning the message processing time. Thus, the variability must be provoked in the transmission of the packets through a given link. In particular, we have further observed that the time spent for the simulated bridge in Cooja has huge variability while forwarding messages from the Cooja environment to the computer and back (The same problem has also been noticed by other Contiki users [43]).

In summary, by means of simulation in Cooja, we have tested variables concerning real deployments, such as the number of nodes and the packet loss ratio. However, the results produced by Cooja are on a different scale than real world values. This is inherent to the Cooja mode of working as a simulator. In particular, while in our real hardware deployment, the (re-)authentication time has been measured in terms of milliseconds, the corresponding Cooja-based results are in seconds. On the other hand, it is worth noting that our Cooja-based simulation results are a good approach to compare the general behavior of our development in terms of a range of users authenticated to network access at the same PAA with communications simulated over constrained networks with different packet loss ratios.

Finally, it is worth noting that a PANA (re-)authentication process in constrained devices implies a PaC and a PRE separated by one hop, which is why our simulations only involve a PaC, a PRE, a bridge and the PAA. Moreover, with the inclusion of additional nodes simulating a multi-hop network, the Cooja bridge implementation causes a huge variability in transmission times.

## 6. Conclusion and Future Work

The network access control solution based on the *Extensible Authentication Protocol* (EAP) and the *Protocol for Carrying Authentication for Network Access* (PANA) is being adopted by Zigbee IP and ETSI Machine-to-Machine (M2M) for constrained environments. Therefore, forthcoming Zigbee sensor devices will include an implementation of EAP/PANA protocols. However, to the best of our knowledge, there are no previously available research studies of the suitability of EAP/PANA solution for these constrained environments. We firmly believe that, although some of the constraints that currently must be handled will disappear as soon as more powerful devices appear, our research work on this current problem area will greatly help researchers to better understand the need for a solution for the current state of constrained networks.

The general purpose definition of EAP/PANA shows a network access control solution proven to work for general purpose computers. In particular, there are open source projects [20,21] implementing these specifications. However, the current specification does not fit the constraints in sensor networks.

In this article, we have analyzed the issue of providing network access control in IoT devices and the use of the standard protocol, PANA, as a promising alternative to transport EAP to allow constrained devices to get authenticated when requiring the sending of information to the Internet.

We have analyzed both the PANA and EAP protocols and propose simplifications without affecting the mandatory parts of the standard. We have focused these analyses on the parts that will be deployed in constrained devices: the PANA client, the EAP peer and the PANA Relay (PRE) function.

In our proposed simplifications, some optional functionalities have been omitted. This may have some implications in terms of operability. In particular, the PAA, which forwards messages between a constrained PaC and the AAA server (*i.e.*, it acts as border router), must follow the policies we have defined in Section 4.7. However, it is worth noting that the PAA could potentially apply a different policy to each PaC authentication, depending on the features available in the actual PaC. In particular, PAA could apply our defined policy when a constrained PaC is being authenticated, while the full functionality policy might be used for authentication of a PaC with more resources.

To corroborate the suitability of our proposed modifications, we deploy a small testbed with real devices to obtain processing times and message sizes, which show some interesting conclusions about the usage of PANA in networks of constrained devices. Furthermore, we set up Cooja-based simulations showing interesting results regarding the variable number of users and different packet loss ratios. Overall, authentication and re-authentication times without using a PRE and with the use of a PRE in real and simulated deployments can be considered reasonable, in contrast to other studies about typical PANA authentication time in non-constrained environments. This shows that PANA, with the simplifications proposed in this paper, can be used for bootstrapping network access control with AAA inter-networking in constrained devices. The major issue is related to message size. The limitations in IEEE 802.15.4, in which the maximum physical layer packet size is 127 octets, means that certain PANA messages (especially when PRE is involved) suffer from fragmentation. This fragmentation increases the measured authentication and re-authentication times. Nevertheless, the impact of fragmentation could be reduced by using some compression algorithm, as proposed in [44]. Furthermore, we ascertain that IEEE 802.15.4g will increase the size of the maximum message length to ≈2 K. With this size, the fragmentation we have observed would disappear, so improving the overall authentication and re-authentication times.

In general, the way of compressing to avoid fragmentation is therefore still an open issue. The authors in [7] already present certain alternatives to compress PANA message format, for example, by removing some unused fields, like reserved fields or padding octets. This area of study will be part of our future work in order to improve PANA deployment in constrained devices.

## Figures and Tables

**Table 1.** Memory sizes. PSK, pre-shared symmetric key.

**#**	**Module**	**ROM (bytes)**	**RAM (bytes)**
1	Contiki (empty main)	65,894	7,560
2	PANA state machine		
2.1	*definition*	2,412	820
2.2	*msg management*	3,360	64
3	EAP state machine		
3.1	*definition*	796	0
3.2	*msg management*	228	0
4	EAP-PSK method	2,568	400
5	AES-CMAC / EAX	11,94	0
6	PANA relay functionality	952	24

	**Total**	77,404	8,868
	*Size added by PANA*	11,510 (14.87 %)	1,308 (14.74 %)

**Table 2.** PANA message length. PaC, PANA Client; PRE, PANA Relay; PAA, PANA Authentication Agent; PAN, PANA Answer; PAR, PANA-Auth-Request (PAR); PNR, PANA-Notification-Request.

**Message direction**	**PANA message**	**PaC** ↔ **PRE**	**PRE** ↔ **PAA**
**AUTHENTICATION**			

**PaC** → **PRE** →**PAA**	**PCI**	16	68
**PaC**← **PRE**← **PAA**	**PAR (S-Flag set)**	40	92 *
**PaC** →**PRE** →**PAA**	**PAN (S-Flag set)**	40	92 *
**PaC**← **PRE**← **PAA**	**PAR (EAP-Request ID, Nonce)**	48	100 *
**PaC** →**PRE** →**PAA**	**PAN (EAP-Response ID, Nonce)**	52	104 *
**PaC**← **PRE**← **PAA**	**PAR (EAP-Request ID)**	32	84
**PaC** →**PRE** →**PAA**	**PAN (EAP-Response ID)**	36	88 *
**PaC**← **PRE**← **PAA**	**PAR (EAP-PSK 1)**	48	100 *
**PaC** →**PRE** →**PAA**	**PAN (EAP-PSK 2)**	84	136 *
**PaC**← **PRE**← **PAA**	**PAR (EAP-PSK 3)**	84	136 *
**PaC** →**PRE** →**PAA**	**PAN (EAP-PSK 4)**	68	120 *
**PaC**← **PRE**← **PAA**	**PAR (EAP-Success)**	88 *	140 *
**PaC** → **PRE** →**PAA**	**PAN (AUTH)**	52	104 *

**RE-AUTHENTICATION**			

**PaC** → **PRE** →**PAA**	**PNR**	40	92 *
**PaC**← **PRE**← **PAA**	**PNA**	40	92 *
**PaC** ← **PRE**← **PAA**	**PAR (EAP-Request ID, Nonce)**	72	124 *
**PaC** → **PRE** →**PAA**	**PAN (EAP-Response ID, Nonce)**	76	128 *
**PaC** ← **PRE**← **PAA**	**PAR (EAP-Request ID)**	56	108 *
**PaC** → **PRE** →**PAA**	**PAN (EAP-Response ID)**	60	112 *
**PaC**← **PRE**← **PAA**	**PAR (EAP-PSK 1)**	72	124 *
**PaC** → **PRE** →**PAA**	**PAN (EAP-PSK 2)**	108 *	160 *
**PaC** ← **PRE**← **PAA**	**PAR (EAP-PSK 3)**	108 *	160 *
**PaC** → **PRE** →**PAA**	**PAN (EAP-PSK 4)**	92 *	144 *
**PaC** ← **PRE**← **PAA**	**PAR (EAP-Success)**	88 *	140 *
**PaC** → **PRE** →**PAA**	**PAN (AUTH)**	52	104 *

**Table 3.** Authentication messages. Mean processing time (ticks).

	**Non-PRE**	**With-PRE**	

	**PaC**	**PaC**	**PRE**
**PCI**	0.99 ± 0.00	1.67 ± 0.13	6.56 ± 0.07
**PAR (S-Flag set)**	4.81 ± 0.04	4.81 ± 0.04	6.50 ± 0.07
**PAN (S-Flag set)**	3.60 ± 0.07	3.52 ± 0.07	12.43 ± 0.11 **
**PAR (EAP-Request ID, Nonce)**	3.06 ± 0.02	3.14 ± 0.03	6.46 ± 0,07
**PAN (EAP-Response ID, Nonce)**	4.03 ± 0.01	3.94 ± 0.02	12.21 ± 0.09 **
**PAR (EAP-Request ID)**	3.04 ± 0.01	3.09 ± 0.02	6.40 ± 0.07
**PAN (EAP-Response ID)**	3.98 ± 0.01	3.86 ± 0.03	12.35 ± 0.11 **
**PAR (EAP-PSK 1)**	3.57 ± 0.06	3.59 ± 0.07	6.51 ± 0.07
**PAN (EAP-PSK 2)**	3.79 ± 0.04	3.74 ± 0.05	12.41 ± 0.10 **
**PAR (EAP-PSK 3)**	4.66 ± 0.06	4.64 ± 0.06	6.63 ± 0.06
**PAN (EAP-PSK 4)**	3.79 ± 0.05	3.68 ± 0.06	12.48 ± 0.11 **
**PAR (EAP-Success)**	3.10 ± 0.02	3.09 ± 0.02	6.68 ± 0.06
**PAN (AUTH)**	5.38 ± 0.06	5.36 ± 0.06	12.47 ± 0.12 **

**Table 4.** Re-authentication mean processing time (ticks) per message.

	**Non-PRE**	**With-PRE**	

	**PaC**	**PaC**	**PRE**
**PNR**	5.96 ± 0.01	5.27 ± 0.08	12.46 ± 0.11 **
**PNA**	3.26 ± 0.05	3.29 ± 0.06	6.50 ± 0.07
**PAR (EAP-Request ID. Nonce)**	3.09 ± 0.02	3.08 ± 0.02	6.59 ± 0.07
**PAN (EAP-Response ID. Nonce)**	4.66 ± 0.06	4.65 ± 0.06	12.46 ± 0.11 **
**PAR (EAP-Request ID)**	3.07 ± 0.02	3.06 ± 0.02	6.53 ± 0.07
**PAN (EAP-Response ID)**	4.55 ± 0.07	4.47 ± 0.07	12.31 ± 0.09 **
**PAR (EAP-PSK 1)**	3.60 ± 0.07	3.58 ± 0.07	6.60 ± 0.07
**PAN (EAP-PSK 2)**	10.63 ± 0.07 **	10.33 ± 0.14 **	12.64 ± 0.11 **
**PAR (EAP-PSK 3)**	4.68 ± 0.06	4.63 ± 0.06	12.45 ± 0.10 **
**PAN (EAP-PSK 4)**	10.26 ± 0.09 **	4.45 ± 0.07	12.68 ± 0.13 **
**PAR (EAP-Success)**	3.11 ± 0.03	3.09 ± 0.02	6.70 ± 0.06
**PAN (AUTH)**	5.33 ± 0.06	5.30 ± 0.06	12.20 ± 0.09 **

## Appendix

### A. Light-Weight PANA State Machine

**Table A1:** Eliminated states in a PaC state machine.

**#**	**State**	**Transition**	**Exit Condition**	**Exit State**
1	ANY	Re-transmissions	rtx_timeout && rtx_counter<rtx_max_num	(no change)

2	ANY	Reach maximum number of transmissions	(rtx_timeout && rtx_counter<rtx_max_num) ∥ sess_timeout	CLOSED

3	ANY except INITIAL	Liveness test initiated by peer	Rx:PNR[P]	(no change)

4	ANY except WAIT-PNA-PING	Liveness test response	Rx:PNA[P]	(no change)

5	INITIAL		Rx:PAR[S] && PAR.exist_avp(“EAP-Payload) && eap_piggyback()	INITIAL
		PAA-initiated handshake optim.	Rx:PAR[S] && PAR.exist_avp(“EAP-Payload) && !eap_piggyback()	WAIT-EAP-MSG
			EAP-RESPONSE	WAIT-PAA

6	WAIT_PAA	PAR-PAN exchange	Rx:PAR[] && !eap_piggyback()	WAIT-EAP-MSG
			Rx:PAN[]	WAIT-PAA

7	WAIT-EAP-MSG	Return PAN/PAR from EAP	EAP-RESPONSE && !eap_piggyback()	WAIT-PAA
			EAP-RESP-TIMEOUT && eap_piggyback()	WAIT-PAA

8	OPEN	Re-authentication initiated by PAA	Rx:PAR[]	WAIT-EAP-MSG
		Session termination initiated by PAA	Rx:PTR[]	CLOSED

9	WAIT-PNA-REAUTH	Session termination initiated by PAA	Rx:PTR[]	CLOSED

10	WAIT-PNA-PING	Re-authentication initiated by PAA	Rx:PAR[]	WAIT-EAP-MSG
		Session termination initiated by PAA	Rx:PTR[]	CLOSED

11	SESS-TERM	Session termination initiated by PaC	Rx:PTA[]	CLOSED
